# A clarion call or a swan song? Commentary: A crisis in comparative psychology: where have all the undergraduates gone?

**DOI:** 10.3389/fpsyg.2015.01867

**Published:** 2015-12-02

**Authors:** Craig F. Bielert, Andrew C. Gallup

**Affiliations:** Psychology Department, State University of New York at OneontaOneonta, NY, USA

**Keywords:** comparative psychology, animal behavior, comparative cognition, evolutionary psychology, ethology

In a recent opinion article, Abramson (2015) focused scrutiny upon the current status of comparative psychology. Once a thriving sub-discipline in psychology, Abramson describes what seems to be a field in peril and offers suggestions to enhance the presence of comparative psychology at the undergraduate level. He points out that currently only one textbook carries the title “Comparative Psychology,” which was last released in 2008. Perhaps even more telling of the current status of the field is that the vast majority of popular introductory psychology texts (~70%) fail in even mentioning this topic in their coverage. Abramson also emphasizes that the number of graduate programs specifically carrying the moniker of comparative psychology are two in number, which perhaps matches the need given the decreasing emphasis on this sub-discipline at the undergraduate level. While many of Abramson's individual observations could be taken as cautionary, when combined they appear as a death knell to the field.

As pessimistic as this message sounds, it may just represent an adaptation to a changing scholarly environment. Currently, if one searches on Amazon.com utilizing the advanced search for books, “animal behavior” brings up 42,362 listings. If one examines the first one hundred of these, seventeen textbooks come up. It seems the field of animal behavior is holding on in place of comparative psychology, though its refugium lies primarily within the disciplines of biology and zoology. The goal of animal behavior reflects an appreciation of Niko Tinbergen's four-pronged approach to understanding the behaviors shown by a species (Tinbergen, 1963), which is evident in most popular textbooks. Therefore, in some respects the approach espoused by those who pioneered the field of comparative psychology is simply being practiced under a different guise.

A careful look into the popular and influential comparative psychology textbooks even 50 years ago suggest there was already a progressing shift in the focus of the field (Hinde, 1966; Denny and Ratner, 1970). An unfortunate consequence of shift from comparative psychology to animal behavior, however, is that many psychology students miss out on exposure to animal behavior due to restrictions in their ability to take advanced courses outside their home department. Therefore, Abramson's point to the lack of psychology undergraduates studying comparative aspects of behavior is certainly warranted. Furthermore, many textbooks under the title “animal behavior” give short shrift to humans and their behavior. This omission is a costly one and would leave those interested in a uniform and inclusive interpretive framework lacking. The loss of a truly encompassing comparative psychology should indeed be viewed as regressive. Comparative psychology made a unique contribution to those behavioral sciences focused on the behavior of our own species, and it has been demonstrated that there is a conceptual benefit from comparing humans with other animals in an all-encompassing theoretic (Nettle, 2009).

While one could voice regret about the loss of a field that provided a comprehensive framework to studying behavior across organisms, the apparent decline of comparative psychology has been potentially balanced by the emergence of new sub-disciplines in psychology. For example, comparative cognition and evolutionary psychology provide similar frameworks for studying cognition and behavior, and in some ways their growth has served to fill the void of courses and programs explicitly labeled comparative psychology. Both fields utilize experimental analyses to infer mechanisms of behavior in ethological perspective. In particular, comparative cognition is quite similar to comparative psychology aside from restricting its research questions and measures to those specific to information processing (Shettleworth, 2010). Evolutionary psychology offers the strengths of an explicit evolutionary perspective to humans, with a primary focus on behavior, though its emphasis on comparative literature is relatively limited in scope. Nonetheless, courses in evolutionary psychology appear to improve student knowledge in evolutionary theory at a greater level than do Introductory Biology classes that emphasize evolution (Short and Hawley, 2014), and thus likely provide psychology undergraduates with many of the benefits that follow a curriculum in comparative psychology. Taken together, the topical coverage of comparative cognition and evolutionary psychology overlaps greatly and extends well beyond the bounds of a comparative psychology course.

Despite the apparent lack of coursework and formal training in “Comparative Psychology,” research in this field is not in short supply. This can be evidenced by the increased number of journals devoted to publishing research taking a comparative perspective to behavior and cognition. However, it is clear that with time research in comparative psychology has become increasingly more interdisciplinary. For example, under the scope of this journal, *Frontiers in Comparative Psychology*, it states “This section encompasses the theoretical, observational and empirical aspects of disciplines including animal learning, animal cognition, behavioral ecology, cognitive science, ethology, evolutionary biology, developmental psychology, endocrinology, neurobiology and behavioral genetics.” The *Journal of Comparative Psychology* welcomes papers on topics that are even more widespread. Certainly the inclusion of such diverse areas of study encompassed all within the umbrella of “Comparative Psychology” suggests the field is not in crisis, but instead is alive and well and simply manifested in a different form.

In summary, we agree with Abramson in the need to unite in the maintenance and development of comparative and evolutionary approaches to the study of behavior, specifically within the field of psychology. However, attempts to explicitly hold onto the name “Comparative Psychology” seem largely fruitless, and perhaps greater traction could be made by modifying curricular constraints to enhance the enrollment of psychology undergraduates within existing courses in animal behavior. Instead of lamenting the past and trying to retain a specific branding, we should adapt to the changing academic landscape, embrace emerging sub-disciplines, and celebrate the contributions of comparative psychology in making the field of behavioral science more encompassing and interdisciplinary. Therefore, Abramson's call may indeed be a swan song.

## Conflict of interest statement

The authors declare that the research was conducted in the absence of any commercial or financial relationships that could be construed as a potential conflict of interest.
